# Prevalence of Goitre in Isfahan, Iran, Fifteen Years After Initiation of Universal Salt Iodization

**DOI:** 10.3329/jhpn.v28i4.6041

**Published:** 2010-08

**Authors:** Aminorroaya Ashraf, Massoud Amini, Silva Hovsepian

**Affiliations:** ^1^ Internal Medicine and Endocrinology, Isfahan Endocrine and Metabolism Research Center, Department of Internal Medicine, Isfahan University of Medical Sciences, Isfahan, Iran; ^2^ Isfahan Endocrine and Metabolism Research Center, Isfahan University of Medical Sciences, Isfahan, Iran

**Keywords:** Autoimmunity, Cross-sectional studies, Goitre, Hypothyroidism, Hyperthyroidism, Impact studies, Iodine, Iodine deficiency, Iran

## Abstract

This cross-sectional study investigated the prevalence of goitre in Isfahan, a centrally-located city in Iran, 15 years after the initiation of universal salt iodization. In total, 2,523 Isfahani adults (1,275 males, 1,248 females) aged >20 years were selected by multi-stage cluster-sampling method. Goitre rate, serum thyroid-stimulating hormone (TSH), thyroxine (T4), thyroid peroxidase antibody (TPOAb), thyroglobulin antibody (TgAb), and urinary iodine concentration (UIC) were measured and compared between the goitrous (n=478) and the non-goitrous (n=2,045) participants. The total goitre rate was 19% (n=478) of the 2,523 adults. The rate of Grade I and II goitre was 12.4% (n=312) and 6.6% (n=166) respectively. The total goitre rate, Grade I and II goitre were more prevalent among women than among men. Hypothyroidism was observed in 6.4% (130/2,045) and 18.6% (89/478) of the non-goitrous and goitrous participants respectively [odds ratio (OR)=3.6, 95% confidence interval (CI) 2.7-4.9, p=0.001]. Hyperthyroidism was present in 0.8% (17/2,045) and 5.2% (29/478) of the non-goitrous and goitrous adults respectively (OR=9.0, 95% CI 4.9-16.6, p=0.001). Hypothyroidism was more prevalent in Grade II than in Grade I goitre and among those without goitre (31.3%, 14.1%, and 6.4% respectively) (p=0.001). Positive TPOAb was observed in 24% (n=50) of the non-goitrous and 33.5% (n=84) of the goitrous subjects (p=0.03). Positive TPOAb was observed in 24.6% (35 of 142) of the Grade I and 45% (49 of 109) of the Grade II goitrous adults (p=0.001). Positive TgAb was observed in 21.6% (n=45) of the non-goitrous and 35.9% (n=90) of the goitrous adults (p=0.001). Positive TgAb was observed in 30.3% (43 of 142) of the Grade I and 43.1% (47 of 109) of the Grade II goitrous adults (p=0.04). The median UIC was 18 μg/dL (range 1-80 μg/dL). It was 17.9 μg/dL and 19 μg/dL in the non-goitrous and goitrous adults respectively. After 15 years of successful universal salt iodization in Isfahan, goitre is still endemic, which may be due to thyroid autoimmunity. However, other environmental or genetic factors may have a role.

## INTRODUCTION

An estimated 750 million people worldwide are at a risk of iodine-deficiency disorders (IDDs), including endemic goitre, hypothyroidism, endemic cretinism, and congenital anomalies (1,2). About 20 million people in Iran suffered from iodine deficiency in 1989 (3) when the national salt-iodization programme was initiated. Despite a comprehensive IDD-control programme, by 1994, less than 50% of rural households consumed iodized salt. Therefore, a law for the mandatory production of iodine salt for households was passed in 1994. Two years after the law was implemented, Azizi *et al*. evaluated the status of iodine intake in 26 provinces of Iran. They concluded that Iran had reached a sustainable control programme for iodine deficiency (4) as more than 90% of households were consuming iodized salt. However, goitre was endemic in all the provinces, although the majority were Grade I goitre (4). The prevalence of goitre and the median urinary iodine concentration (UIC) in Isfahan province, a centrally-located city in Iran, was reported to be 40-50% and 13-20 μg/dL respectively in 1994 (4).

The principal indicator of universal salt iodization for the control of IDDs is the median UIC, and the second indicator is thyroid size. It reflects by the prevalence of goitre (1) and the relationship between iodine intake and thyroid disease which is U-shaped. It means that both high and low iodine intake is associated with thyroid diseases, especially the presence of excessive iodine which accelerates thyroid autoimmunity disorders (5,6).

In Tehran, Heydarian *et al*. investigated the rate of goitre, autoimmunity, and UIC before and after the salt-iodization programme. Results of the study showed that the prevalence of goitre decreased significantly in 1999-2000, about 5-6 years after starting the programme. They concluded that salt iodization resulted in adequate UIC, a decrease in serum thyroid-stimulating hormone (TSH) and subclinical hypothyroidism in males, and an increase in thyroid autoantibodies without any significant change in thyroid abnormalities (7).

The aim of our study was to assess the effects of iodine supplementation on the prevalence of goitre, UIC, and thyroid autoantibodies among adults of Isfahan, Iran, with a population of 1,986,542 (male 1,017,940, female 968,602) in 2006, 15 years after the salt-iodization programme.

## MATERIALS AND METHODS

### Study population

In a cross-sectional study, 2,600 Isfahani adults were selected by multi-stage cluster-sampling method. At first stage, we randomly selected 40 blocks on the city map. Then, we asked the post office to give us the addresses of all the homes in each block. We randomly selected 960 addresses from the list supplied by the post office (24 homes in each block). In this way, 2,600 adults were invited. In total, 2,523 (97%) of the invited adults, aged 20-86 years, accepted our invitation and came for examination and testing from January to April 2006. Their mean age was 39 [standard deviation (SD) 12.4] years. Of the 2,523 adults, 1,275 were males (50.5%), and 1,248 were females (49.5%).

Trained personnel informed the people door to door and invited them to enroll into the study. People were asked to come to the Isfahan Endocrine and Metabolism Research Center (IEMRC), according to a pre-planned appointment. Seven trained general practitioners filled out a questionnaire to provide some demographic data (sex, age, education, employment status, and home address), past history of thyroid disorders, past or current usage of medications, including iodine supplementations (vitamins), iodine overload (amiodarone), or other drugs that could interfere with thyroid function (glucococorticoids). They checked the past medical documents of the participants and recorded the past medical history, including thyroid disorders, for each person.

Those who had any history of thyroid disorders or abnormalities on physical examination or laboratory finding (TSH <0.3 mIU/L or TSH >4 mIU/L) were recalled for visit by the endocrinologist (AA). We sent a letter to each person. On this recall letter, the TSH concentration of the participants was sent to them by express mail to make sure that they can keep it as a medical document in their medical files to show to their own physician, if they would like. The interval between the first examination and the recalled invitation was about 2-3 weeks. 

### Assessment of goitre

Thyroid size was graded according to the classification of the World Health Organization to Grade 0 (no palpable or visible), goitre I (palpable but not visible), and goitre II (visible) (2). The sum of Grade I and II was considered the total goitre rate (TGR) of the population studied.

### Serum TSH, T4, T3, and thyroid autoantibody assays

Blood samples were obtained from all the participants and urine samples from about one-fourth (n=710) of them. Collected serum and urine samples were both frozen at-20°C in the IEMRC laboratory.

In those people who had high TSH (>4 mIU/L) or low TSH (<0.3 mIU/L) concentration, the second blood sample was taken to measure TSH, T4, T3, and T3RU. Free T4 index (FT4I) was calculated by T4*T3RU. The second blood sample was taken at the same day from those who accepted our recall invitation and visited by the endocrinologist. It was about four weeks after the time in which the first blood sample had been taken.

TSH was measured by IRMA (immunoradiometric assay) (Kavoshyar kits, Tehran, Iran). Intra-assay and inter-assay CV was 1.5% and 1.9% respectively. The normal range for TSH was 0.3-4 μU/mL.

Serum T4 was assayed by radioimmunoassay (RIA) (Kavoshyar kits, Tehran, Iran). Its intra- and inter-assay CV was 4.7% and 4.9% respectively. The normal range for T4 concentration was 4.5-12 μg/dL. The normal range for T3 concentration was 80-190 ng/dL. T3RU was assayed by RIA. Its intra- and inter-assay CV was 3.6% and 4.4% respectively. The normal range for T3RU concentration was 25-35%. The normal range for FT4I according to our laboratory was 1.3-4.8.

TgAb and TPOAb were measured by Rapid ELISA (Genesis Diagnostic Co., London, UK). Intra-assay and inter-assay CV for TgAb was less than 12%, and for TPOAb, it was 7% and 5% respectively. Positive TgAb and TPOAb were considered to be concentrations more than 100 IU/mL and 75 IU/mL, respectively.

### Measurement of urinary iodine

UIC was measured by the digestion method based on a modification of Sandell-Kolthoff reaction. The intra-assay and inter-assay CV was 1.25% and 2.2% respectively (8). UIC of <10 μg/dL was considered iodine deficiency, UIC of >30 μg/dL iodine excess, and in between iodine sufficiency (1).

*Definitions*: Thyroid function was defined as euthyroid (TSH level within the normal range, 0.3-4 mIU/L), overt hypothyroidism (TSH >4 mIU/L and low FT4I levels), subclinical hypothyroidism (TSH level >4 mIU/L and normal serum FT4I levels), overt hyperthyroidism (TSH level <0.3 mIU/L and high FT4I or high T3), and subclinical hyperthyroidism (TSH level <0.3 mIU/L and normal FT4I and normal T3) (8).

### Statistical analysis

Data were analyzed using the SPSS software (version 13) and the Epi Info software (version 6.04). Variables with normal distribution, such as age, were expressed as mean (SD). To compare the mean age in different grades of goitre, analysis of variance (ANOVA) was used. To figure out where the difference existed when ANOVA showed that there was a difference between means in these groups, Scheffe test was applied. Those variables whose distribution was not normal were expressed as median (range). To compare the median of TSH, TPOAb, TgAb, and UIC, the presence of thyroid dysfunction (hypothyroidism, hyperthyroidism), thyroid nodule, positive antithyroid antibodies (TPOAb, TgAb) between non-goitrous subjects and those with Grade I and Grade II goitre, chi-square test was used. The prevalence of goitre among different age-groups (≤30, 31-40, 41-50, and >50 years), sex-groups, and according to UIC and thyroid autoantibodies was compared by chi-square test. The p values of <0.05 were considered significant.

### Ethics

Our study was conducted in accordance with the ethical standards of the IEMRC Committee on Human Experimentation and with the Helsinki Declaration. The Regional Committee for Ethics of IEMRC and Isfahan University of Medical Sciences approved the study. Consents were obtained from participants before recruitment to the study.

## RESULTS

The mean age of the 2,523 adults studied was 39 (SD 12.4) years (range 20-86 years). Of them, 1,275 were males, and 1249 were females. The mean age of the males was 41 (SD 12.7) years (range 20-80 years), and the mean age of the females was 37 (SD 12.4) years (range 20-86 years).

The TGR was present in 19% (478/2,523) of the study subjects. The rate of Grade I and II goitre was 12.4% (312/2,523) and 6.6% (166/2,523) respectively. The prevalence of TGR, Grade I and II goitre was higher among women than among men (28.8%, 17.3%, and 11.5% for women vs 9.2%, 7.5%, and 1.7% for men (p<0.05). The prevalence of TGR, Grade I and II goitre among adults according to their age-groups and gender is presented in Table 1. The rate of goitre decreased significantly (p<0.05) with the increase in age. Goitre was observed in 14.3% of the menopausal women (224/1,245) (7.6% Grade I, 6.7% Grade II), which was lower compared to non-menopausal women (324/1,006) [(14.3% vs 321%, odds ratio (OR)=0.35, 95% confidence interval (CI) 0.24-0.52, p=0.001)].

**Table 1.** Prevalence of total goitre rate, Grade I and II goitre among adult Isfahani population: according to age-groups and gender in 2006

Age-group (years)	Grade I		Grade II		TGR	
	np/n	%	np/n	%	np/n	%
Male						
≤30	29/308	9.4	6/308	1.9	35/308	11.3
31-40	32/323	9.9	9/323	2.8	41/323	12.7
41-50	22/394	5.6	5/394	1.3	27/394	6.9
>50	11/250	6.1	2/250	1.1	13/250	7.2
Total	94/1,275		22/1,275	116/1,275	
Female						
≤30	97/448	21.7	52/448	11.6	149/448	33.3
31-40	55/319	17.2	50/319	15.7	105/319	32.9
41-50	49/273	17.9	26/273	9.5	75/273	27.4
>50	13/208	8.0	12/208	7.4	25/208	15.4
Total	214/1,249		140/1,249		354/1,248	
All						
≤30	126/756	16.7	58/756	7.7	184/756	24.4
31-40	87/642	13.5	59/642	9.2	146/642	22.7
41-50	71/667	10.6	31/667	4.6	102/667	15.2
>50	24/458	5.2	14/458	3.1	38/458	8.3
Total	308/2,523		162/2,523		470/2,523	

np=Number of patients with goitre;

n=Number of adults studied;

TGR=Total goitre rate

Characteristics of the non-goitrous and goitrous study population (Grade I and II and TGR) are reported in Table 2.

**Table 2.** Comparison of characteristics of goitrous (TGR*, Grade I and II) and non-goitrous Isfahani adults in 2006 [median (range)] or [mean (SD)]

Parameter	Non-goitrous (n=2,045)	Grade I (n=312)	Grade II (n=166)	TGR (n=478)	p value
Age (years)	39.9 (12.6)	35.1 (10.8)	36.3 (10.6)	35.5 (10.8)	0.001
TSH (mU/L)	1.8 (0.04-127)	2 (0.03-100)	2.3 (0.02-83)	2 (0.02-100)	0.02
TPOAb (IU/mL)	6.8 (0.2-78) (n=208)	8.2 (0-3,000) (n=142)	42 (0-3,000) (n=109)	15.2 (0-3,000) (n=251)	0.001
TgAb (IU/mL)	13.5 (0-9,000) (n=208)	24.5 (0-8,430) (n=142)	84 (0-9,000) (n=109)	54 (0-9,000) (n=251)	0.001
UIC (μg/dL)	17.9 (1–80) (n=454)	17.7 (2.9-80) (n=162)	20.6 (2–80) (n=94)	19 (2–80) (n=256)	0.1

*TGR: Total goitre rate (Grade I and II);

SD=Standard deviation;

TgAb=Thyroglobulin antibody;

TPOAb=Thyroid peroxidase antibody;

TSH=Thyroid-stimulating hormone;

UIC=Urinary iodine concentration

Clinical nodule was observed in 2.5% of the adults. Thyroid nodule was observed in 9.4% of the goitrous patients [Grade I (5.1%) and II (17.5%) goitre] versus 0.8% of the non-goitrous population (OR=12.4, 95% CI 7.0-21.9, p=0.001). It was observed that 5.1% of the adults had Grade I goitre and 17.5% had Grade II goitre. Clinical nodule was more prevalent among the non-goitrous women than among men (1.2% vs 0.5%, OR=2.4, 95% CI 0.9-6.5, p=0.007) but it was similar in the goitrous females (9.4%) and males (9.3%).

### Thyroid function and goitre

Hypothyroidism was present in 6.4% (130/2,045) and 18.6% (89/478) of the non-goitrous and goitrous participants respectively (OR=3.6, 95% CI 2.7-4.9, p=0.001). Whereas hyperthyroidism was observed in 0.8% (17/2,045) and 5.2% (29/478) of the non-goitrous and goitrous participants respectively (OR=9.0, 95% CI 4.9-16.6, p=0.001). Hypothyroidism was more prevalent in subjects with Grade II than in Grade I goitre and those without goitre (31.3%, 14.1%, and 6.4% respectively) (p=0.001).

### Goitre according to TSH level

The prevalence of goitre (TGR), according to low, normal and high TSH levels, was 6.7% (n=21), 77.2% (n=241), and 16.0% (n=50) respectively. TGR was more prevalent among subjects with higher than with low TSH level (p<0.05).

The prevalence of Grade I goitre, according to low, normal and high TSH levels, was 9.2% (n=44), 70.9% (n=339), and 19.9% (n=95) respectively. The prevalence of Grade II goitre, according to low, normal and high TSH levels, was 13.9% (n=23), 59% (n=98), and 27.1% (n=45) respectively.

### Goitre and thyroid autoantibodies

Thyroid autoantibodies were measured in 459 subjects (208 non-goitrous, 251 goitrous). Table 3 shows the prevalence of positive antibodies among the goitrous and non-goitrous people in Isfahan. TPOAb and TgAb were positive in 29.2% (134/459) and 29.4% (135/459) of the adults. It was not higher in females (28.4%) than in males (31.1%) (p=0.32).

**Table 3.** Prevalence of positive thyroid autoantibodies among goitrous and non-goitrous people in Isfahan, 2006

Goitre status	TPOAb or TgAb		TPOAb and TgAb		Only TgAb		Only TPOAb	
	No.	%	No.	%	No.	%	No.	%
Non-goitrous (n=208)	68	32.7	27	13	45	21.6	50	37.3
Goitrous (n=251)								
Grade I	58/142	40.8	20/142	14.1	43/142	30.3	35/142	24.6
Grade II	68/109	62.4**	28/109	25.7**	47/109	43.1**	49/109	44.9**
TGR	126	50.2†	46	18.3†	90	35.8†	84	33.5†

**p<0.05 Grade II vs non-goitrous in each antibody category with chi-square test;

†p<0.05 TGR vs non-goitrous in each antibody category with chi-square test;

TgAb=Thyroglobulin antibody;

*TGR: Total goitre rate (Grade I and II);

TPOAb=Thyroid peroxidase antibody

Positive TPOAb was present in 24% (n=50) of the non-goitrous and 33.5% (n=84) of goitrous subjects (p=0.03). Positive TPOAb was observed in 24.6% (35 of 142) of the Grade I goitrous and 45% (49 of 109) of the Grade II goitrous participants (p=0.001).

TgAb was positive in 21.6% (n=45) of the non-goitrous and 35.9% (n=90) of the goitrous subjects (p=0.001). TgAb was positive in 30.3% (43/142) of people with Grade I goitre and 43.1% in those with (47 of 109) of Grade II goitre patients (p=0.04).

### Goitre and urinary iodine concentration

UIC was measured in 710 participants (454 non-goitrous, 256 goitrous). The median UIC was 18 μg/dL (range 1-80 μg/dL) for the study participants. It was 17.9 μg/dL and 19 μg/dL in the non-goitrous and goitrous subjects respectively [not significant (NS)].

Iodine deficiency, sufficiency, and excess was observed in 152 (21.4%), 425 (59.9%), and 133 (18.7%) of the adults respectively (p<0.01).

In the goitrous patients, 23% (n=59), 55.5% (n=149), and 21.5% (n=55) had iodine deficiency, sufficiency, and excess respectively (p=NS). There was no significant difference between the UIC and the prevalence of goitre (p=NS).

## DISCUSSION

This study investigated the prevalence of goitre among the adults aged 20 years and older of Isfahan city, 15 years after the implementation of the national salt-iodization programme. Considering that iodine deficiency was resolved in this city (median of UIC is 18 μg/dL), it is supposed that the prevalence of goitre must have been decreased. Although the rate of goitre decreased significantly, it is still endemic (19%). However, the prevalence of visible goitre was not high (6.6%) which is a good success in decreasing goitre.

Our findings corroborate with those of other studies in our country, which indicate that goitre persists in their study areas, despite adequate supplementation of iodine (7,9–10). In their study in Shahriar among 3,146 subjects aged 3-70 years, Azizi *et al*. reported that 12 years after the initiation of universal salt iodization in Iran, its prevalence was still high (47%), although the rate of goitre decreased significantly (9). Heydarian *et al.* reported that goitre was observed in 33% of women and 15.5% of men, 10 years after the implementation of salt-iodization programme (10). In another study in Tehran, the rates of goitre, thyroid function, and autoantibodies in adults aged ≥20 years were compared before 1983-1984 and after 1999-2000 national salt iodization. The prevalence of goitre was 25.2% (15.5% Grade I and 9.7% Grade II) in the study population with adequate iodine intake (7). It was 65.2% before the supplementation of iodine.

Comparing our results with those of the above-mentioned studies, the prevalence of goitre in Isfahan was lower. Our survey was done 15 years after iodine supplementation. Therefore, taking adequate iodine for a longer time is expected to decrease the size of goitre more efficiently. The longer duration of living in an iodine-sufficient state could explain the lower rate of goitre. However, it is still high. Either the time to decrease the size of goitre is not enough, or other factors could have a role.

A study in Laos to assess the impact of salt-iodization programme found a higher median value of urinary iodine but almost a similar prevalence of goitre after the introduction of iodinated salt compared to an earlier period (11).

In Iran, salt iodization of 40 parts per million (PPM) has been done since 1994. The type of iodine has been used is potassium iodide. At regular intervals, adequacy of iodine supplementation has routinely been assessed in factories, shops, and households by the Ministry of Health. It has been adequate. For example, at factories and households, the mean (SD) iodine salt content has been about 33 (11) ppm and 32 (11) ppm respectively. However, adequacy of salt iodization has been well-monitored (12).

Recently, in their second national report on monitoring iodine-deficiency control, Azizi *et al*. have reported a marked reduction in the rate of goitre (9.8%) and adequate UIC in school children (12). They concluded that the prevalence of goitre decreased some years after the normalization of UIC, and for adults and older individuals, it may even take longer to achieve the effect of salt iodization (12).

In our survey, the age of the adults ranged from 20 to 80 years, and the salt-iodization programme has been in place for 15 years before the study was conducted. It is expected that the full effect of iodine supplementation on goitre rate would be observed among those whose whole life has been spent in this period of time (children and adolescents) and not in older people whose established goitre is still persistent at least in some degrees.

The prevalence of goitre in our country increased with age and was higher among women than among men as reported in other studies (7,10). In the HUNT study in Norway, which has been considered an iodine-sufficient area, the prevalence of goitre was 2.9% among women and 0.4% among men, and it was lower among younger groups than among older groups (13).

The median UIC in this study showed iodine sufficiency (UIC=18 μg/dL), and the prevalence of TGR, Grade I and II goitre was not different among subgroups with iodine deficiency, sufficiency, and excess. Although iodine excess was observed in 18.7% of the participants, it did not have any correlation between goitre and iodine excess in the present study. It can be explained by the fact that urinary iodine excretion measured in one urine sample reflects the state of iodine intake just at that special day. However, data of other previous studies in Iran support our findings in being no correlation between UIC and goitre rate (7,10).

The two studies in India conducted among adolescents reported that there was no correlation between the iodine status and the goitre grades too (14,15).

Thus, our findings (Table 2 and 3) raise the issue with regard to whether the high prevalence of goitre in this region is due to autoimmunity similar to what has already been reported by Heydarian *et al.* in Tehran (7). However, according to many studies, iodine is implicated in triggering or enhancing thyroid autoimmunity, at least in the formerly iodine-deficient areas. This phenomenon has also been observed in other countries which were previously iodine-deficient where the transition to sufficient or excessive iodine intake was followed by an increase in the incidence of thyroid autoimmunity (16,17). However, some other studies found no supporting evidence of autoimmunity induction after iodine administration to correct iodine deficiency (18,19).

The presence of thyroid autoantibodies indicates an autoimmune thyroid disease component that may lead to the development of thyroid dysfunction.

In our study, the prevalence of positive TPOAb and TgAb was higher among people with visible goitre than among those without goitre (Table 3). It confirms the role of autoimmunity for Grade II goitre in the Isfahani adults.

In a clinic-based study in Isfahan, 10-12 years after iodine repletion, the prevalence of positive thyroid autoantibodies was studied among women with and without thyroid diseases; the rate of positive TPOAb/TgAb in women with simple goitre was 48.9% and was much higher than the control group (35.6%) (20). Our population-based study supports the findings of that clinic-based report.

In our study, most goitrous adults were euthyroid. However, goitre was more prevalent among people with thyroid dysfunction.

According to several studies, when iodine intake is changing from deficiency to sufficiency, there may be more cases of autoimmune hypothyroidism whereas the increase in iodine intake may, over time, lead to hyperthyroidism (21).

In this study, thyroid nodule was more prevalent among the goitrous than among non-goitrous subjects, especially those with Grade II goitre. It increased with age. In the study of Heydarian *et al.*, it did not increase with age (10).

Isfahan is an iodine-replete area. However, goitre is still endemic after 15 years of sufficient iodine intake. It seems that thyroid autoantibodies have a major role in the pathogenesis of goitre. Other probable environmental factors, such as selenium, iron and vitamin A deficiency, or thiocyanate overload, and genetic factors, should be investigated (22–24). Repetition of the study in the next decade is also suggested.

## ACKNOWLEDGEMENTS

The authors thank the study participants for their cooperation and the field staff, including Dr. Zahra Nezhadnik, Dr. Azamosadat Tabatabaei, Dr. Sima Beheshti, Dr. Shadab Shateri, Dr. Zahra Fallah, Dr. Marjan Momenzadeh, Dr. Mahnaz Soghrati, Dr. Hayedeh Adilipour, and Dr. Hamid Reza Sirus, for their assistance in gathering data and Mr. Majid Abyar for his technical computer support.
